# Genomic Contributors to Rhythm Outcome of Atrial Fibrillation Catheter Ablation – Pathway Enrichment Analysis of GWAS Data

**DOI:** 10.1371/journal.pone.0167008

**Published:** 2016-11-21

**Authors:** Daniela Husser, Petra Büttner, Laura Ueberham, Borislav Dinov, Philipp Sommer, Arash Arya, Gerhard Hindricks, Andreas Bollmann

**Affiliations:** Department of Electrophysiology, Heart Center Leipzig, Leipzig University, Leipzig, Germany; University of Minnesota, UNITED STATES

## Abstract

**Background:**

Left atrial enlargement and persistent atrial fibrillation (AF) are well-known predictors for arrhythmia recurrence after AF catheter ablation (LRAF). In this study, by using pathway enrichment analysis of GWAS data, we tested the hypothesis that genetic pathways associated with these phenotypes are also associated with LRAF.

**Methods:**

Samples from 660 patients with paroxysmal (n = 370) or persistent AF (n = 290) undergoing de-novo AF catheter ablation were genotyped for ~1,000,000 SNPs. SNPs found to be significantly associated with left atrial diameter (LAD) or AF type were used for gene-based association tests in a systematic biological Knowledge-based mining system for Genome-wide Genetic studies (KGG). Associated genes were tested for pathway enrichment using WEB-based Gene SeT AnaLysis Toolkit (WebGestalt), the Gene Annotation Tool to Help Explain Relationships (GATHER) and the databases provided by Kyoto Encyclopedia of Genes and Genomes (KEGG). In a second step, the association of consistently enriched pathways and LRAF was tested.

**Results:**

By using sequential 7-day Holter ECGs, LRAF between 3 and 12 months was observed in 48% and was associated with LAD (B = 1.801, 95% CI 0.760–2.841, p = 1.0E-3) and persistent AF (OR = 2.1; 95% CI 1.567–2.931, p = 2.0E-6). WebGestalt (adj. p = 2.7E-22) and GATHER (adj. p = 5.2E-3) identified the calcium signaling pathway (hsa04020) as the only consistently enriched pathway for LAD, while the extracellular matrix (ECM) -receptor interaction pathway (hsa04512) was the only consistently enriched pathway for AF type (adj. p = 2.1E-15 in WebGestalt; adj. p = 9.3E-4 in GATHER). Both calcium signaling (adj. p = 2.2E-17 in WebGestalt; adj. p = 2.9E-2 in GATHER) and ECM-receptor interaction (adj. p = 1.2E-10 in WebGestalt; adj. p = 2.9E-2 in GATHER) were significantly associated with LRAF.

**Conclusions:**

Calcium signaling and ECM-receptor interaction pathways are associated with LAD and AF type and, in turn, with LRAF. Future and larger studies are necessary to replicate and apply these findings.

## Introduction

Genetic studies have revealed diverse mechanisms of atrial fibrillation (AF), the most common cardiac arrhythmia [1]. This heterogeneous pathophysiology may—at least in part—explain the limited efficacy of different rhythm control strategies. Among those, catheter ablation is an established treatment modality for AF, but arrhythmia recurrence is also observed in up to 50% of patients within 1 year after ablation [2]. A classification system that recognizes AF subtypes based on culprit genes and/or clinical data has the potential to guide treatment strategies [3]. In fact, recent candidate-gene studies have linked common genetic variants with rhythm outcome after AF ablation [4,5]. Previous work has also consistently identified left atrial enlargement and persistent AF as clinical predictors for ablation success [2]. However, whether or not, the genetic background of those predictive clinical variables modulates risk for arrhythmia recurrence is unknown.

Pathway-based analysis of GWAS data is a powerful tool to detect subtle but systematic patterns in the genome that underpin complex diseases, natural disease progression and responses to therapy. For instance, this approach has been successfully applied to identify novel regulatory pathways in different phenotypes such as body mass index [6], colorectal cancer [7] or outcome of breast cancer [8].

Here, for the first time, we use pathway enrichment analysis of GWAS data to test the hypothesis that genetically-modulated pathways associated with left atrial enlargement and persistent AF also associate with arrhythmia recurrence following AF catheter ablation.

## Methods

### Patients

Six hundred-and-sixty AF patients undergoing de-novo radiofrequency AF catheter ablation between 2008 and 2013 were enrolled in the Leipzig Heart Center AF ablation registry. Paroxysmal AF was defined as self-terminating episodes of AF within 7 days after onset documented by ECG or an ambulatory ECG monitor. Persistent AF was defined as an AF episode either lasting longer than 7 days or requiring drug or direct current cardioversion for termination.

In all patients, transthoracic and transesophageal echocardiography was performed prior to catheter ablation. Left atrial diameter (LAD) and left ventricular ejection fraction were determined using standard measurements and a left atrial thrombus was excluded. All class I or III antiarrhythmic medications with the exception of amiodarone were discontinued at least 5 half-lives before the procedure.

The study protocol was approved by the Ethics Committee of the Leipzig University Medical Faculty. All patients signed written informed consent for study participation. All methods were performed in accordance with the relevant guidelines and regulations.

### AF catheter ablation and follow-up

Left atrial catheter ablation was performed using a previously described approach [4]. In brief, patients were studied under deep propofol sedation with continuous invasive monitoring of arterial blood pressure and oxygen saturation. Non-fluoroscopic 3D catheter orientation, CT image integration, and tagging of the ablation sites were performed using Ensite NavX, Ensite Velocity (St. Jude Medical, St. Paul, MN, USA) or CARTO 3 (Biosense Webster, Diamond Bar, CA, USA). Trans-septal access and catheter navigation were performed with a steerable sheath (Agilis, St. Jude Medical, St. Paul, MN, USA). Patients presenting with AF at the beginning of the procedure were electrically cardioverted and ablation was performed during sinus rhythm (i.e. AF termination by ablation was not attempted). In all patients circumferential left atrial ablation lines were placed around the antrum of the ipsilateral pulmonary veins (irrigated tip catheter, pre-selected tip temperature of 48°C, and maximum power of 20–40 W). In patients with persistent AF, empiric linear lesions were added at the left atrial roof, the basal posterior wall and the left atrial isthmus or in low voltage areas.

After circumferential line placement, voltage and pace mapping along the ablation line were used to identify and close gaps. The isolation of all pulmonary veins with bidirectional block was verified with a multipolar circular mapping catheter and was defined as the procedural endpoint.

After ablation, class I and III antiarrhythmic drugs were not reinitiated. Oral anticoagulation was prescribed for 6 months, and proton pump inhibitors were added for 4 weeks. All patients were followed in the outpatient clinic for 12 months after the ablation. During this follow-up period, 7-day Holter ECG recordings were performed 3, 6 and 12 months after the ablation. Additional ECGs and Holter ECG recordings were obtained when patients’ symptoms were suggestive of AF. AF recurrence was defined as a documented atrial arrhythmia episode (AF and/or atrial tachycardia, AT) lasting longer than 30 seconds between 3 and 12 months after the ablation (thus, including a 3-month “blanking period”). All patients with sustained early recurring AF underwent direct cardioversion. Additional drug administration was left to the discretion of the treating physician.

### Sample processing

Blood samples were obtained in EDTA test tubes in fasting state prior ablation. Genomic DNA was isolated using a commercial kit according to the manufacturer’s recommendations (PeqLab, Erlangen, Germany). Genotyping was performed using HumanOmniExpressExome-8-v1.2 arrays comprising about one million Single Nucleotide Polymorphisms (SNPs) according to established protocols (Illumina, San Diego, US).

### Data analysis and statistics

Raw data was compiled using GenomeStudio (Illumina) software and exported to PLINK GWAs analysis package [9]. Using PLINK tool set the data was tested for consistency. Samples with a call rate <95% were excluded. Single SNPs had to meet the following criteria: minor allele frequencies (MAF) > 0.01, call rate > 95%, Hardy-Weinberg equilibrium (HWE) significance threshold > 0.0001. Otherwise they were excluded from further analysis.

Association of genotypes with LAD was detected using linear regression with adjustment for age, gender and AF type. Association of genotypes with AF type (persistent AF) and arrhythmia recurrence was detected using logistic regression analysis with adjustment for age and gender. All variants identified by this approach were included in the analysis under an additive genetic model without testing for recessive or dominant genetic effects.

Illumina specific “exmSNPs” were assigned to their corresponding dbSNP rs IDs. The resulting SNP lists were used for gene enrichment. This was done with Knowledge-based mining system for Genome-wide Genetic studies (KGG) [10]. R-square values representing linkage disequilibrium data corresponding to the CEU (Northern Europeans from Utah) population was received from 1000 Genomes project phase 1v3 to adjust for SNP dependency. SNPs were mapped onto genes according to GenCode v23 information’s. SNPs within a range of 5kb upstream and downstream of the gene were assigned to the gene. If a SNP was in the overlapping region of two genes it was assigned to both. The KGG GATES algorithm, an extension of Simes test, was used to calculate enrichment p-values incorporating functional SNP weights controlling for LD and gene length. Enrichment p-values < 0.05 were regarded statistically significant.

For pathway enrichment analysis we used the Gene Annotation Tool to Help Explain Relationships (GATHER) [11] and WEB-based Gene SeT AnaLysis Toolkit (WebGestalt) [12] together with the databases provided by Kyoto Encyclopedia of Genes and Genomes (KEGG) [13]. Non-random over representation of genes from our candidate gene list in specific KEGG pathways was regarded significant when Fisher's exact test p-value with False Discovery Rate (GATHER) or hypergeometric distribution p-value corrected for multiple testing using Bonferroni correction (WebGestalt) was < 0.05.

**Fig 1:**
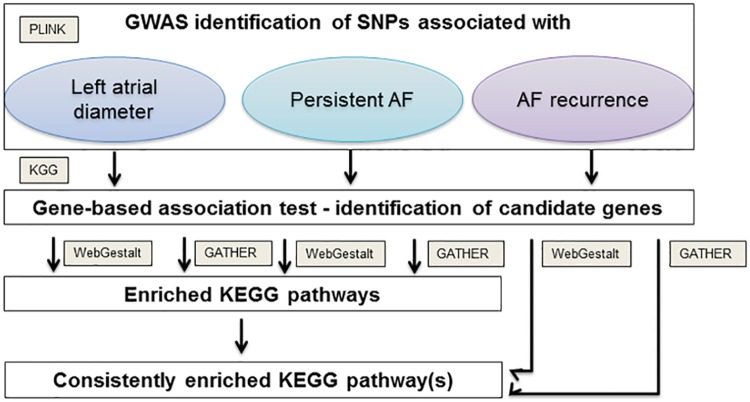
Flow chart of pathway enrichment analysis based on GWAS of LAD, AF type and AF recurrence after catheter ablation.

We applied a two-stage analysis plan. First, we identified consistently enriched KEGG pathways in LAD and AF type present in both enrichment tools. Second, association of those identified pathway(s) with arrhythmia recurrence was tested with both enrichment tools (Fig 1).

Clinical variables are presented as mean +/- one standard deviation or percentages. They were compared between patients with and without AF recurrence using chi-square or Student’s t-test.

## Results

### Patient characteristics

The study population included 660 patients with a history of paroxysmal (n = 370) or persistent AF (n = 290, Table 1). Arrhythmia recurrence between 3 and 12 months was observed in 48% (AF only in 69%, AT only in 23%, AF/AT in 8%) and was associated with LAD (B = 1.801, 95% CI 0.760–2.841, p = 1.0E-3) and persistent AF (OR = 2.1; 95% CI 1.567–2.931, p = 2E-6) in multivariate analysis.

**Table 1:** Patient characteristics.

	Total* (n = 660)	AF recurrence (n = 318)	No recurrence (n = 341)	p value
Age (years)	60 ± 10	61 ± 10	59 ± 10	7.55E-03
Male gender (%)	68	70	67	ns
Idiopathic AF (%)	14	15	12	ns
Persistent AF (%)	44	53	35	1.00E-06
LAD (mm)	43 ± 6	44 ± 6	42 ± 6	5.42E-05
LVEF (%)	59 ± 10	58 ± 10	59 ± 9	ns

* One patient was lost to follow-up

Genotyping call rate in all subjects was > 95% except in three samples (<85%) that were excluded from further analysis.

### Pathways associated with left atrial diameter and persistent AF

28.062 SNPs were associated with LAD and were annotated to 10.252 genes while 27.400 SNPs were associated with AF type and were annotated to 10.180 genes. Of those, 1.778 SNPs and 3.834 genes were found in both phenotypes.

In WebGestalt, 101 KEGG pathways were associated with LAD and 91 with persistent AF (S1 and S2 Tables), while 77 were associated with both phenotypes. Of those, statistical significant associations in both enrichment tools were identified for calcium signaling pathway (hsa04020) and LAD as well as extracellular matrix (ECM) -receptor interaction (hsa04512) and persistent AF (Table 2).

**Table 2:** Calcium signaling and ECM-receptor interaction pathways and their association with LAD, AF type and arrhythmia recurrence using two enrichment tools.

Enrichment tool	Phenotype	C	O	E	R	rawP	adjP
**Calcium signaling**							
WebGestalt	LAD	177	85	27.12	3.13	1.2E-24	2.7E-22
	LRAF	177	77	26.62	2.89	9.7E-20	2.2E-17
GATHER	LAD	196	88			4.0E-05	5.2E-03
	LRAF	196	81			4.3E-04	2.9E-02
**ECM-receptor interaction**							
WebGestalt	AF type	85	47	12.78	3.68	9.3E-18	2.1E-15
	LRAF	85	41	12.78	3.21	5.1E-13	1.2E-10
GATHER	AF type	86	45			7.2E-06	9.3E-04
	LRAF	86	41			4.2E-04	2.9E-02

C, the number of reference genes in the category; O, the number of genes in the gene set and also in the category; E, expected number in the category; R, the ratio of enrichment, rawP, the p value from hypergeometric test (WebGestalt) or Fisher exact test (GATHER); adjP, the p value adjusted by the multiple test adjustment.

Please note that GATHER does not provide E and R.

### Pathways and genes associated with AF recurrence

**Fig 2:**
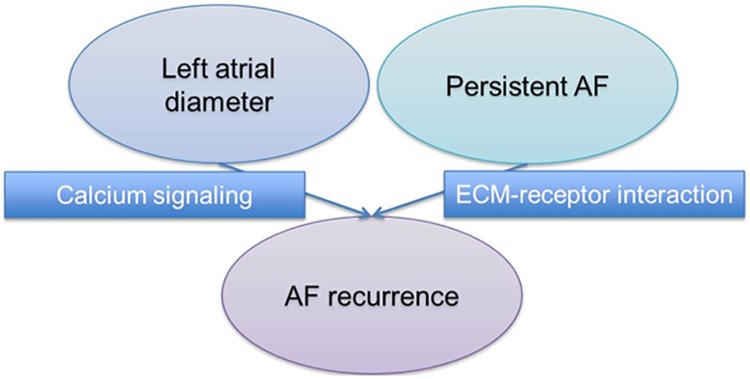
Clinical and genomic associations between LAD, AF type and AF recurrence. Left atrial enlargement and persistent AF are associated with rhythm outcome of AF catheter ablation, but whether or not there are shared common genetic pathways is unknown. In this study, we found calcium signaling and ECM-receptor interaction pathways to be associated with left atrial diameter and persistent AF and, in turn, with AF recurrences.

Both calcium signaling (adj. p = 2.2E-17 in WebGestalt; adj. p = 2.9E-2 in GATHER) and ECM-receptor interaction (adj. p = 1.2E-10 in WebGestalt; adj. p = 2.9E-2 in GATHER) were significantly associated with rhythm outcome (Table 2, Fig 2).

Within the calcium signaling pathway, there were 55 genes that were associated with LAD and LRAF while there were 26 genes in ECM-receptor interaction pathway that were associated with persistent AF and LRAF (Tables 3 and 4).

**Table 3:** Genes of the calcium signaling pathway associated with LAD and LRAF (in alphabetical order).

Gene	Full name	LAD	LRAF
*ADCY2*	adenylate cyclase 2	1,8E-02	4,8E-02
*ADCY3*	adenylate cyclase 3	3,4E-02	2,6E-02
*ADCY8*	adenylate cyclase 8	1,4E-02	4,4E-02
*ADRA1A*	adrenoceptor alpha 1A	1,4E-02	3,1E-02
*ATP2B2*	ATPase, Ca++ transporting, plasma membrane 2	8,3E-03	4,0E-02
*BDKRB2*	bradykinin receptor B2	1,2E-02	2,3E-02
*CACNA1A*	calcium channel, voltage-dependent, P/Q type, alpha 1A subunit	3,0E-02	1,2E-02
*CACNA1B*	calcium channel, voltage-dependent, N type, alpha 1B subunit	4,2E-02	2,5E-02
*CACNA1C*	calcium channel, voltage-dependent, L type, alpha 1C subunit	7,8E-03	6,2E-03
*CACNA1D*	calcium channel, voltage-dependent, L type, alpha 1D subunit	7,6E-03	2,4E-02
*CACNA1E*	calcium channel, voltage-dependent, R type, alpha 1E subunit	8,7E-03	4,0E-03
*CACNA1H*	calcium channel, voltage-dependent, T type, alpha 1H subunit	3,8E-02	1,6E-02
*CACNA1S*	calcium channel, voltage-dependent, L type, alpha 1S subunit	2,5E-02	2,7E-02
*CALML3*	calmodulin-like 3	4,8E-02	2,2E-02
*CAMK2A*	calcium/calmodulin-dependent protein kinase II alpha	1,6E-02	3,3E-02
*CAMK4*	calcium/calmodulin-dependent protein kinase IV	6,3E-03	3,0E-04
*CHRM2*	cholinergic receptor, muscarinic 2	2,0E-02	4,4E-02
*CHRM3*	cholinergic receptor, muscarinic 3	1,9E-02	1,5E-02
*CREBBP*	CREB binding protein	2,0E-02	1,5E-02
*EGFR*	epidermal growth factor receptor	5,0E-03	2,4E-02
*ERBB4*	v-erb-a erythroblastic leukemia viral oncogene homolog 4	2,3E-02	4,9E-02
*GNA14*	guanine nucleotide binding protein, alpha 14	3,9E-02	1,9E-02
*GNAL*	guanine nucleotide binding protein, alpha activating activity polypeptide, olfactory type	7,9E-04	3,2E-02
*GNAQ*	guanine nucleotide binding protein, q polypeptide	4,3E-02	4,9E-03
*GRIN1*	glutamate receptor, ionotropic, N-methyl D-aspartate 1	3,3E-02	9,3E-03
*GRM1*	glutamate receptor, metabotropic 1	5,0E-02	4,2E-02
*GRM5*	glutamate receptor, metabotropic 5	2,3E-02	1,9E-02
*HRH2*	histamine receptor H2	1,6E-03	4,9E-02
*HTR2A*	5-hydroxytryptamine receptor 2A, G protein-coupled	4,9E-02	4,7E-02
*ITPR1*	inositol 1,4,5-trisphosphate receptor, type 1	3,5E-02	1,1E-02
*ITPR2*	inositol 1,4,5-trisphosphate receptor, type 2	1,9E-02	3,3E-02
*ITPR3*	inositol 1,4,5-trisphosphate receptor, type 3	8,4E-03	4,2E-02
*LHCGR*	luteinizing hormone/choriogonadotropin receptor	1,6E-02	8,5E-03
*NFATC1*	nuclear factor of activated T-cells, cytoplasmic, calcineurin-dependent 1	4,5E-02	8,7E-03
*NFATC2*	nuclear factor of activated T-cells, cytoplasmic, calcineurin-dependent 2	3,9E-02	6,2E-03
*P2RX6*	purinergic receptor P2X, ligand-gated ion channel, 6	4,4E-02	2,7E-02
*PDGFRB*	platelet-derived growth factor receptor, beta polypeptide	1,2E-02	2,5E-02
*PLCB1*	phospholipase C, beta 1	2,1E-03	3,4E-02
*PLCB4*	phospholipase C, beta 4	4,4E-02	4,7E-02
*PLCE1*	phospholipase C, epsilon 1	2,3E-02	4,6E-02
*PLCG2*	phospholipase C, gamma 2	2,2E-02	7,5E-03
*PRKCA*	protein kinase C, alpha	3,6E-02	4,8E-02
*PRKCB*	protein kinase C, beta	4,4E-02	3,3E-02
*PTGER3*	prostaglandin E receptor 3	3,3E-02	4,2E-02
*PTGFR*	prostaglandin F receptor	2,2E-02	5,0E-02
*PTK2B*	PTK2B protein tyrosine kinase 2 beta	6,6E-03	1,9E-02
*PYGL*	phosphorylase, glycogen, liver	4,4E-02	3,6E-02
*RYR1*	ryanodine receptor 1	4,7E-02	2,7E-02
*RYR2*	ryanodine receptor 2	3,9E-02	4,1E-02
*RYR3*	ryanodine receptor 3	2,0E-02	4,4E-02
*SLC8A1*	solute carrier family 8, member 1	2,1E-02	3,0E-02
*SLC8A3*	solute carrier family 8, member 3	3,0E-02	4,5E-02
*SYK*	spleen tyrosine kinase	4,0E-03	4,1E-02
*TACR3*	tachykinin receptor 3	4,8E-02	3,3E-02
*TTN*	titin	1,5E-02	4,6E-02

**Table 4:** Genes of the ECM-receptor interaction pathway associated with AF type and LRAF (in alphabetical order).

Gene	Full name	AF type	LRAF
*AGRN*	agrin	1,2E-02	2,5E-02
*CD36*	CD36 antigen	2,1E-02	5,3E-03
*COL4A2*	collagen, type IV, alpha 2	1,6E-02	8,7E-03
*COL4A4*	collagen, type IV, alpha 4	3,9E-02	3,4E-02
*COL5A1*	collagen, type V, alpha 1	3,7E-02	4,6E-03
*COL5A3*	collagen, type V, alpha 3	4,2E-02	2,0E-02
*DAG1*	dystroglycan 1	9,3E-03	1,3E-02
*FN1*	fibronectin 1	4,2E-02	4,2E-02
*HSPG2*	heparan sulfate proteoglycan 2	4,9E-02	1,6E-02
*ITGA1*	integrin, alpha 1	4,7E-02	3,9E-02
*ITGA4*	integrin, alpha 4	2,2E-02	2,3E-02
*ITGA9*	integrin, alpha 9	1,9E-02	3,1E-03
*ITGB3*	integrin, beta 3	2,0E-02	2,6E-02
*ITGB4*	integrin, beta 4	3,7E-02	1,9E-02
*ITGB5*	integrin, beta 5	1,4E-02	4,0E-02
*ITGB6*	integrin, beta 6	3,4E-02	2,5E-02
*LAMA1*	laminin, alpha 1	2,2E-02	4,0E-02
*LAMA3*	laminin, alpha 3	4,5E-02	1,7E-02
*LAMA5*	laminin, alpha 5	1,3E-02	3,3E-02
*LAMC3*	laminin, gamma 3	3,6E-02	1,1E-02
*RELN*	reelin	4,9E-02	3,7E-02
*SDC2*	syndecan 2	3,5E-03	2,7E-02
*SV2B*	synaptic vesicle glycoprotein 2B	2,3E-02	1,5E-02
*TNR*	tenascin R	4,7E-02	9,6E-03
*TNXB*	tenascin XB	2,7E-02	2,4E-02
*VWF*	von Willebrand factor	4,9E-02	2,6E-02

## Discussion

### Main findings

This study is the first to explore shared genetic pathways of predictive clinical variables such as left atrial enlargement and AF type and response to AF catheter ablation. In a two-stage association study, we first identified calcium signaling and ECM-receptor interaction as common regulatory pathways for LAD and persistent AF in two enrichment tools. In a second, candidate-based step, those pathways were found to also associate with rhythm outcome of AF catheter ablation in both tools. This analysis approach is novel and the identification of the genomic background of those pathways and its link to clinical variables and response to therapy is a relevant finding.

### Clinical and genetic predictors of AF ablation outcomes

While left atrial enlargement and AF persistence have been linked with AF ablation outcome [2], atrial fibrosis is an emerging prognostic marker. In fact, atrial tissue fibrosis estimated by delayed enhancement MRI was independently associated with likelihood of recurrent arrhythmia [14]. Moreover, several plasma markers of fibrotic turnover have been related with rhythm outcome [15]. Interestingly, genetic polymorphisms implicated in central profibrotic and inflammatory pathways such as SNPs of the angiotensin-converting enzyme gene (*ACE*), [16] the angiotensinogen gene (*AGT*) [17] or the interleukin-6 receptor gene (*IL6R*) [18] have also been found to predict recurring atrial arrhythmias after AF ablation.

In this study, we associated calcium signaling and ECM-receptor interaction pathways with left atrial dilatation and AF persistence and subsequently with rhythm outcome.

### Calcium signaling and ECM-receptor interaction pathway genes and AF

Interestingly, some of the calcium signaling and ECM-receptor interaction pathway genes found in our study (Tables 3 and 4) have been shown to exert effects on AF development and AF progression. For instance, gain-of-function mutations in *RyR2* have been shown to predispose to catecholaminergic polymorphic ventricular tachycardia and AF by enhanced propensity for spontaneous Ca(2+) release [19]. In addition, familial and early-onset AF have been linked with rare variants of two CACNA genes with overlapping effects on the Cav1.2 (encoded by *CACNA1C*) [20] or junctophilin 2 (*JPH2*) resulting in defective RyR2-mediated sarcoplasmatic reticulum Ca(2+) release [21]. Abnormal sarcoplasmic reticulum Ca(2+) leak via ryanodine receptor type 2 (RyR2) has been observed as a source of ectopic activity [22], the hallmark of AF initiation. Moreover, abnormal calcium signaling is also implicated in atrial fibrosis, the main driver of AF maintenance and progression. Ca(2+) influx into atrial fibroblasts induces proliferation and differentiation into collagen-secreting myofibroblasts and subsequently heterogeneous conduction slowing and reentry [23]. Angiotensin-II is an important contributor to AF-related remodeling. Inositol 1,4,5-trisphosphate receptors have been shown to mediate angiotensin-1 receptor associated Ca(2+) release [24].

*ITGA9* encodes an alpha integrin, an integral membrane glycoprotein that mediates diverse functions including cell–cell and cell–matrix adhesion, proliferation, and apoptosis and is associated with the PR interval on the surface ECG [25]. PR interval prolongation in turn has been linked with advanced atrial remodeling and worse ablation outcome [26].

Very recently, thrombin has been found to cause pro-fibrotic and pro-inflammatory responses in adult atrial fibroblasts and promote the development of a substrate for AF [27]. Thrombin generation is, however, sensitive to von Willebrand (vWF) factor activity [28] that is at least in part modulated by the *vWF* gene [29].

In summary, these data together with our findings strongly suggest the involvement of calcium signaling and ECM-receptor interaction pathway genes in AF-remodeling associated phenotypes such as left atrial enlargement and AF persistence which then in turn may serve as markers for AF ablation rhythm outcome.

### Limitations

Our study is based on small sample size. We addressed this by using well-defined intermediate AF phenotypes that are known markers of AF ablation outcome and different bioinformatics tools. Moreover, we required the genotype—phenotype correlation to be present in two different pathway enrichment tools and we used a two-stage analysis approach; (1) unselected identification of consistently enriched pathways and (2) selected “candidate-”pathway analysis for association with outcome. Single SNPs were not in the center of the study rather we focused on the most significant candidate genes from enrichment analysis. Consequently, candidate genes and pathways with lower significance levels or failing our stringent identification process could have been overlooked by this approach. In addition, we did not consider dominant or recessive SNP effects that should be done in future studies. Single pathway components were not assessed in detail that was beyond the scope of this study. Finally, ablation approaches in persistent AF are evolving and linear lesion sets have lessened in popularity due to no incremental benefit and even pro-arrhythmia. This may impact on type of arrhythmia recurrence and should be considered when assessing recurrence rates and comparing this to other studies.

### Conclusions

Calcium signaling and ECM-receptor interaction pathways are associated with LAD and AF type and, in turn, with LRAF. Future and larger studies are necessary to replicate and apply these findings.

## Supporting Information

S1 Table KEGG pathways associated with LAD using WebGestalt (in alphabetical order). (DOC)

S2 Table KEGG pathways associated with AF type using WebGestalt (in alphabetical order). (DOC)
