# Supplementary material for: Genomic Contributors to Rhythm Outcome of Atrial Fibrillation Catheter Ablation – Pathway Enrichment Analysis of GWAS Data
Source: PLoS One. 2016 Nov 21;11(11):e0167008. doi: 10.1371/journal.pone.0167008 (PMC5117760; doi:10.1371/journal.pone.0167008)
Supplement: S1 Table — (DOC) [file pone.0167008.s001.doc]

**S1 Table.**

| **KEGG pathway** | **C** | **O** | **E** | **R** | **rawP** | **adjP** |
| --- | --- | --- | --- | --- | --- | --- |
| ABC transporters | 44 | 24 | 6,74 | 3,56 | 2,0E-09 | 4,6E-07 |
| Acute myeloid leukemia | 57 | 24 | 8,73 | 2,75 | 1,1E-06 | 3,0E-04 |
| Adherens junction | 73 | 33 | 11,19 | 2,95 | 1,3E-09 | 3,0E-07 |
| Adipocytokine signaling pathway | 68 | 28 | 10,42 | 2,69 | 2,6E-07 | 5,9E-05 |
| Aldosterone-regulated sodium reabsorption | 42 | 26 | 6,44 | 4,04 | 8,3E-12 | 1,9E-09 |
| alpha-Linolenic acid metabolism | 20 | 11 | 3,06 | 3,59 | 4,7E-05 | 1,1E-02 |
| Alzheimer's disease | 167 | 53 | 25,59 | 2,07 | 8,1E-08 | 1,8E-05 |
| Amoebiasis | 106 | 43 | 16,24 | 2,65 | 3,2E-10 | 7,3E-08 |
| Amyotrophic lateral sclerosis (ALS) | 53 | 20 | 8,12 | 2,46 | 5,8E-05 | 1,3E-02 |
| Arachidonic acid metabolism | 59 | 23 | 9,04 | 2,54 | 8,9E-06 | 2,0E-03 |
| Arrhythmogenic right ventricular cardiomyopathy (ARVC) | 74 | 40 | 11,34 | 3,53 | 1,4E-14 | 3,1E-12 |
| Axon guidance | 129 | 69 | 19,77 | 3,49 | 9,6E-24 | 2,2E-21 |
| B cell receptor signaling pathway | 75 | 28 | 11,49 | 2,44 | 2,7E-06 | 6,0E-04 |
| Bacterial invasion of epithelial cells | 70 | 24 | 10,73 | 2,24 | 6,8E-05 | 1,6E-02 |
| beta-Alanine metabolism | 22 | 12 | 3,37 | 3,56 | 2,4E-05 | 5,4E-03 |
| Bile secretion | 71 | 42 | 10,88 | 3,86 | 3,5E-17 | 8,0E-15 |
| Calcium signaling pathway | 177 | 85 | 27,12 | 3,13 | 1,2E-24 | 2,7E-22 |
| Carbohydrate digestion and absorption | 44 | 22 | 6,74 | 3,26 | 7,7E-08 | 1,8E-05 |
| Cardiac muscle contraction | 77 | 31 | 11,8 | 2,63 | 1,1E-07 | 2,5E-05 |
| Cell adhesion molecules (CAMs) | 133 | 56 | 20,38 | 2,75 | 1,1E-13 | 2,6E-11 |
| Chagas disease (American trypanosomiasis) | 104 | 33 | 15,94 | 2,07 | 2,1E-05 | 4,8E-03 |
| Chemokine signaling pathway | 189 | 59 | 28,96 | 2,04 | 3,0E-08 | 6,9E-06 |
| Chronic myeloid leukemia | 73 | 26 | 11,19 | 2,32 | 1,6E-05 | 3,7E-03 |
| Complement and coagulation cascades | 69 | 26 | 10,57 | 2,46 | 5,0E-06 | 1,1E-03 |
| Cytokine-cytokine receptor interaction | 265 | 80 | 40,61 | 1,97 | 7,0E-10 | 1,6E-07 |
| Dilated cardiomyopathy | 90 | 44 | 13,79 | 3,19 | 7,8E-14 | 1,8E-11 |
| Dorso-ventral axis formation | 24 | 15 | 3,68 | 4,08 | 1,9E-07 | 4,4E-05 |
| ECM-receptor interaction | 85 | 38 | 13,03 | 2,92 | 1,1E-10 | 2,6E-08 |
| Endocytosis | 201 | 67 | 30,8 | 2,18 | 1,7E-10 | 3,8E-08 |
| Endometrial cancer | 52 | 21 | 7,97 | 2,64 | 1,1E-05 | 2,6E-03 |
| ErbB signaling pathway | 87 | 36 | 13,33 | 2,7 | 4,5E-09 | 1,0E-06 |
| Ether lipid metabolism | 36 | 15 | 5,52 | 2,72 | 1,0E-04 | 2,3E-02 |
| Fat digestion and absorption | 46 | 18 | 7,05 | 2,55 | 7,8E-05 | 1,8E-02 |
| Fatty acid metabolism | 43 | 20 | 6,59 | 3,04 | 1,3E-06 | 3,0E-04 |
| Fc epsilon RI signaling pathway | 79 | 32 | 12,11 | 2,64 | 5,9E-08 | 1,3E-05 |
| Fc gamma R-mediated phagocytosis | 94 | 37 | 14,41 | 2,57 | 1,4E-08 | 3,2E-06 |
| Focal adhesion | 200 | 86 | 30,65 | 2,81 | 6,5E-21 | 1,5E-18 |
| Gap junction | 90 | 38 | 13,79 | 2,76 | 8,5E-10 | 1,9E-07 |
| Gastric acid secretion | 74 | 39 | 11,34 | 3,44 | 8,8E-14 | 2,0E-11 |
| Glioma | 65 | 29 | 9,96 | 2,91 | 1,8E-08 | 4,2E-06 |
| Glycerolipid metabolism | 50 | 21 | 7,66 | 2,74 | 5,4E-06 | 1,2E-03 |
| Glycerophospholipid metabolism | 80 | 30 | 12,26 | 2,45 | 1,1E-06 | 2,0E-04 |
| Glycosaminoglycan biosynthesis - heparan sulfate | 26 | 13 | 3,98 | 3,26 | 3,7E-05 | 8,3E-03 |
| GnRH signaling pathway | 101 | 43 | 15,48 | 2,78 | 4,9E-11 | 1,1E-08 |
| Hedgehog signaling pathway | 56 | 20 | 8,58 | 2,33 | 1,0E-04 | 2,3E-02 |
| Hepatitis C | 134 | 46 | 20,53 | 2,24 | 4,1E-08 | 9,4E-06 |
| Hypertrophic cardiomyopathy (HCM) | 83 | 39 | 12,72 | 3,07 | 9,6E-12 | 2,2E-09 |
| Inositol phosphate metabolism | 57 | 24 | 8,73 | 2,75 | 1,1E-06 | 3,0E-04 |
| Insulin signaling pathway | 138 | 49 | 21,15 | 2,32 | 4,3E-09 | 9,7E-07 |
| Intestinal immune network for IgA production | 48 | 20 | 7,36 | 2,72 | 1,1E-05 | 2,4E-03 |
| Jak-STAT signaling pathway | 155 | 48 | 23,75 | 2,02 | 7,2E-07 | 2,0E-04 |
| Leishmaniasis | 72 | 26 | 11,03 | 2,36 | 1,2E-05 | 2,8E-03 |
| Leukocyte transendothelial migration | 116 | 43 | 17,78 | 2,42 | 8,4E-09 | 1,9E-06 |
| Long-term depression | 70 | 44 | 10,73 | 4,1 | 2,2E-19 | 4,9E-17 |
| Long-term potentiation | 70 | 37 | 10,73 | 3,45 | 3,3E-13 | 7,6E-11 |
| Lysine degradation | 44 | 19 | 6,74 | 2,82 | 9,3E-06 | 2,1E-03 |
| Lysosome | 121 | 40 | 18,54 | 2,16 | 9,3E-07 | 2,0E-04 |
| MAPK signaling pathway | 268 | 103 | 41,07 | 2,51 | 2,5E-20 | 5,8E-18 |
| Melanogenesis | 101 | 37 | 15,48 | 2,39 | 1,3E-07 | 2,9E-05 |
| Melanoma | 71 | 29 | 10,88 | 2,67 | 2,0E-07 | 4,5E-05 |
| Metabolic pathways | 1130 | 350 | 173,17 | 2,02 | 4,3E-41 | 9,9E-39 |
| mTOR signaling pathway | 52 | 22 | 7,97 | 2,76 | 2,8E-06 | 6,0E-04 |
| Natural killer cell mediated cytotoxicity | 136 | 42 | 20,84 | 2,02 | 3,7E-06 | 8,0E-04 |
| Neuroactive ligand-receptor interaction | 272 | 108 | 41,68 | 2,59 | 1,6E-22 | 3,6E-20 |
| Neurotrophin signaling pathway | 127 | 42 | 19,46 | 2,16 | 5,0E-07 | 1,0E-04 |
| Non-small cell lung cancer | 54 | 24 | 8,28 | 2,9 | 3,3E-07 | 7,6E-05 |
| Notch signaling pathway | 47 | 18 | 7,2 | 2,5 | 1,0E-04 | 2,3E-02 |
| Oocyte meiosis | 112 | 34 | 17,16 | 1,98 | 4,4E-05 | 1,0E-02 |
| Osteoclast differentiation | 128 | 43 | 19,62 | 2,19 | 2,2E-07 | 5,1E-05 |
| Pancreatic cancer | 70 | 23 | 10,73 | 2,14 | 2,0E-04 | 4,6E-02 |
| Pancreatic secretion | 101 | 48 | 15,48 | 3,1 | 2,4E-14 | 5,4E-12 |
| Pathways in cancer | 326 | 127 | 49,96 | 2,54 | 2,5E-25 | 5,6E-23 |
| Phosphatidylinositol signaling system | 78 | 39 | 11,95 | 3,26 | 8,0E-13 | 1,8E-10 |
| PPAR signaling pathway | 70 | 32 | 10,73 | 2,98 | 1,6E-09 | 3,7E-07 |
| Progesterone-mediated oocyte maturation | 86 | 28 | 13,18 | 2,12 | 5,1E-05 | 1,2E-02 |
| Propanoate metabolism | 32 | 14 | 4,9 | 2,85 | 1,0E-04 | 2,3E-02 |
| Prostate cancer | 89 | 32 | 13,64 | 2,35 | 1,4E-06 | 3,0E-04 |
| Protein digestion and absorption | 81 | 38 | 12,41 | 3,06 | 1,9E-11 | 4,3E-09 |
| Protein processing in endoplasmic reticulum | 165 | 43 | 25,29 | 1,7 | 2,0E-04 | 4,6E-02 |
| Purine metabolism | 162 | 53 | 24,83 | 2,13 | 2,6E-08 | 5,9E-06 |
| Regulation of actin cytoskeleton | 213 | 80 | 32,64 | 2,45 | 1,8E-15 | 4,2E-13 |
| Renal cell carcinoma | 70 | 29 | 10,73 | 2,7 | 1,4E-07 | 3,1E-05 |
| Rheumatoid arthritis | 91 | 37 | 13,95 | 2,65 | 4,9E-09 | 1,1E-06 |
| Salivary secretion | 89 | 41 | 13,64 | 3,01 | 6,4E-12 | 1,5E-09 |
| Small cell lung cancer | 85 | 35 | 13,03 | 2,69 | 8,6E-09 | 2,0E-06 |
| Steroid hormone biosynthesis | 56 | 20 | 8,58 | 2,33 | 1,0E-04 | 2,3E-02 |
| T cell receptor signaling pathway | 108 | 38 | 16,55 | 2,3 | 2,9E-07 | 6,6E-05 |
| TGF-beta signaling pathway | 84 | 26 | 12,87 | 2,02 | 2,0E-04 | 4,6E-02 |
| Thyroid cancer | 29 | 14 | 4,44 | 3,15 | 3,0E-05 | 6,9E-03 |
| Tight junction | 132 | 53 | 20,23 | 2,62 | 4,7E-12 | 1,1E-09 |
| Toxoplasmosis | 132 | 52 | 20,23 | 2,57 | 1,8E-11 | 4,0E-09 |
| Tryptophan metabolism | 42 | 21 | 6,44 | 3,26 | 1,5E-07 | 3,4E-05 |
| Type I diabetes mellitus | 43 | 18 | 6,59 | 2,73 | 2,7E-05 | 6,1E-03 |
| Type II diabetes mellitus | 48 | 24 | 7,36 | 3,26 | 2,0E-08 | 4,5E-06 |
| Ubiquitin mediated proteolysis | 135 | 37 | 20,69 | 1,79 | 2,0E-04 | 4,6E-02 |
| Valine, leucine and isoleucine degradation | 44 | 19 | 6,74 | 2,82 | 9,3E-06 | 2,1E-03 |
| Vascular smooth muscle contraction | 116 | 60 | 17,78 | 3,38 | 7,0E-20 | 1,6E-17 |
| VEGF signaling pathway | 76 | 33 | 11,65 | 2,83 | 4,6E-09 | 1,0E-06 |
| Vibrio cholerae infection | 54 | 20 | 8,28 | 2,42 | 7,9E-05 | 1,8E-02 |
| Viral myocarditis | 70 | 26 | 10,73 | 2,42 | 6,7E-06 | 1,5E-03 |
| Wnt signaling pathway | 150 | 48 | 22,99 | 2,09 | 2,4E-07 | 5,6E-05 |

C, the number of reference genes in the category; O, the number of genes in the gene set and also in the category; E, expected number in the category; R, the ratio of enrichment, rawP, the p value from hypergeometric test; adjP, the p value adjusted by the multiple test adjustment.
