# Supplementary material for: Genomic Contributors to Rhythm Outcome of Atrial Fibrillation Catheter Ablation – Pathway Enrichment Analysis of GWAS Data
Source: PLoS One. 2016 Nov 21;11(11):e0167008. doi: 10.1371/journal.pone.0167008 (PMC5117760; doi:10.1371/journal.pone.0167008)
Supplement: S2 Table — (DOC) [file pone.0167008.s002.doc]

**S2** Table.

| **KEGG pathway** | **C** | **O** | **E** | **R** | **rawP** | **adjP** |
| --- | --- | --- | --- | --- | --- | --- |
| ABC transporters | 44 | 23 | 6.61 | 3.48 | 8.9E-09 | 2.0E-06 |
| Acute myeloid leukemia | 57 | 20 | 8.57 | 2.33 | 1.0E-04 | 2.3E-02 |
| Adherens junction | 73 | 30 | 10.97 | 2.73 | 6.6E-08 | 1.5E-05 |
| Alzheimer's disease | 167 | 49 | 25.10 | 1.95 | 1.8E-06 | 4.0E-04 |
| Amoebiasis | 106 | 43 | 15.93 | 2.70 | 1.7E-10 | 3.9E-08 |
| Amyotrophic lateral sclerosis (ALS) | 53 | 20 | 7.97 | 2.51 | 4.4E-05 | 9.9E-03 |
| Arachidonic acid metabolism | 59 | 24 | 8.87 | 2.71 | 1.7E-06 | 4.0E-04 |
| Arrhythmogenic right ventricular cardiomyopathy | 74 | 43 | 11.12 | 3.87 | 1.8E-17 | 4.1E-15 |
| Axon guidance | 129 | 61 | 19.39 | 3.15 | 4.1E-18 | 9.2E-16 |
| B cell receptor signaling pathway | 75 | 32 | 11.27 | 2.84 | 8.2E-09 | 1.8E-06 |
| Bacterial invasion of epithelial cells | 70 | 30 | 10.52 | 2.85 | 2.1E-08 | 4.7E-06 |
| Bile secretion | 71 | 31 | 10.67 | 2.90 | 7.1E-09 | 1.6E-06 |
| Butirosin and neomycin biosynthesis | 5 | 5 | 0.75 | 6.65 | 7.7E-05 | 1.7E-02 |
| Calcium signaling pathway | 177 | 70 | 26.61 | 2.63 | 1.8E-15 | 4.1E-13 |
| Cardiac muscle contraction | 77 | 25 | 11.57 | 2.16 | 9.7E-05 | 2.2E-02 |
| Cell adhesion molecules (CAMs) | 133 | 61 | 19.99 | 3.05 | 2.6E-17 | 5.9E-15 |
| Chagas disease (American trypanosomiasis) | 104 | 32 | 15.63 | 2.05 | 3.7E-05 | 8.4E-03 |
| Chemokine signaling pathway | 189 | 56 | 28.41 | 1.97 | 2.4E-07 | 5.5E-05 |
| Chronic myeloid leukemia | 73 | 26 | 10.97 | 2.37 | 1.1E-05 | 2.6E-03 |
| Colorectal cancer | 62 | 27 | 9.32 | 2.90 | 7.0E-08 | 1.6E-05 |
| Cytokine-cytokine receptor interaction | 265 | 68 | 39.84 | 1.71 | 4.5E-06 | 1.0E-03 |
| Dilated cardiomyopathy | 90 | 43 | 13.53 | 3.18 | 2.1E-13 | 4.7E-11 |
| Drug metabolism - cytochrome P450 | 73 | 26 | 10.97 | 2.37 | 1.1E-05 | 2.6E-03 |
| ECM-receptor interaction | 85 | 47 | 12.78 | 3.68 | 9.3E-18 | 2.1E-15 |
| Endocytosis | 201 | 76 | 30.22 | 2.52 | 2.1E-15 | 4.8E-13 |
| Endometrial cancer | 52 | 19 | 7.82 | 2.43 | 1.0E-04 | 2.3E-02 |
| ErbB signaling pathway | 87 | 38 | 13.08 | 2.91 | 1.5E-10 | 3.3E-08 |
| Ether lipid metabolism | 36 | 16 | 5.41 | 2.96 | 2.4E-05 | 5.3E-03 |
| Fat digestion and absorption | 46 | 17 | 6.91 | 2.46 | 2.0E-04 | 4.5E-02 |
| Fc epsilon RI signaling pathway | 79 | 31 | 11.88 | 2.61 | 1.4E-07 | 3.2E-05 |
| Fc gamma R-mediated phagocytosis | 94 | 41 | 14.13 | 2.90 | 3.0E-11 | 6.6E-09 |
| Focal adhesion | 200 | 86 | 30.06 | 2.86 | 1.8E-21 | 4.1E-19 |
| Galactose metabolism | 27 | 12 | 4.06 | 2.96 | 2.0E-04 | 4.5E-02 |
| Gap junction | 90 | 31 | 13.53 | 2.29 | 3.8E-06 | 9.0E-04 |
| Gastric acid secretion | 74 | 25 | 11.12 | 2.25 | 4.7E-05 | 1.1E-02 |
| Glioma | 65 | 24 | 9.77 | 2.46 | 1.2E-05 | 2.7E-03 |
| Glycerolipid metabolism | 50 | 24 | 7.52 | 3.19 | 3.7E-08 | 8.4E-06 |
| Glycerophospholipid metabolism | 80 | 34 | 12.03 | 2.83 | 3.2E-09 | 7.2E-07 |
| Glycine, serine and threonine metabolism | 32 | 15 | 4.81 | 3.12 | 2.0E-05 | 4.4E-03 |
| Glycolysis / Gluconeogenesis | 65 | 22 | 9.77 | 2.25 | 1.0E-04 | 2.3E-02 |
| Glycosaminoglycan biosynthesis - heparan sulfate | 26 | 12 | 3.91 | 3.07 | 2.0E-04 | 4.5E-02 |
| GnRH signaling pathway | 101 | 39 | 15.18 | 2.57 | 6.3E-09 | 1.4E-06 |
| Hematopoietic cell lineage | 88 | 31 | 13.23 | 2.34 | 2.2E-06 | 5.0E-04 |
| Hypertrophic cardiomyopathy (HCM) | 83 | 38 | 12.48 | 3.05 | 2.6E-11 | 5.9E-09 |
| Inositol phosphate metabolism | 57 | 24 | 8.57 | 2.80 | 7.8E-07 | 2.0E-04 |
| Insulin signaling pathway | 138 | 47 | 20.74 | 2.27 | 2.1E-08 | 4.8E-06 |
| Intestinal immune network for IgA production | 48 | 18 | 7.22 | 2.49 | 1.0E-04 | 2.3E-02 |
| Leishmaniasis | 72 | 23 | 10.82 | 2.13 | 2.0E-04 | 4.5E-02 |
| Leukocyte transendothelial migration | 116 | 41 | 17.44 | 2.35 | 5.1E-08 | 1.1E-05 |
| Linoleic acid metabolism | 30 | 13 | 4.51 | 2.88 | 2.0E-04 | 4.5E-02 |
| Long-term depression | 70 | 37 | 10.52 | 3.52 | 1.8E-13 | 4.1E-11 |
| Long-term potentiation | 70 | 32 | 10.52 | 3.04 | 9.9E-10 | 2.2E-07 |
| MAPK signaling pathway | 268 | 101 | 40.29 | 2.51 | 7.6E-20 | 1.7E-17 |
| Melanogenesis | 101 | 30 | 15.18 | 1.98 | 1.0E-04 | 2.3E-02 |
| Melanoma | 71 | 24 | 10.67 | 2.25 | 6.5E-05 | 1.5E-02 |
| Metabolic pathways | 1130 | 326 | 169.87 | 1.92 | 2.2E-33 | 5.0E-31 |
| Metabolism of xenobiotics by cytochrome P450 | 71 | 26 | 10.67 | 2.44 | 6.4E-06 | 1.4E-03 |
| Natural killer cell mediated cytotoxicity | 136 | 40 | 20.44 | 1.96 | 1.4E-05 | 3.2E-03 |
| Neuroactive ligand-receptor interaction | 272 | 104 | 40.89 | 2.54 | 6.1E-21 | 1.4E-18 |
| Neurotrophin signaling pathway | 127 | 43 | 19.09 | 2.25 | 1.0E-07 | 2.3E-05 |
| Non-small cell lung cancer | 54 | 20 | 8.12 | 2.46 | 6.0E-05 | 1.4E-02 |
| Notch signaling pathway | 47 | 20 | 7.07 | 2.83 | 5.3E-06 | 1.2E-03 |
| Oocyte meiosis | 112 | 37 | 16.84 | 2.20 | 1.5E-06 | 3.0E-04 |
| Osteoclast differentiation | 128 | 40 | 19.24 | 2.08 | 2.8E-06 | 6.0E-04 |
| Pancreatic secretion | 101 | 45 | 15.18 | 2.96 | 1.3E-12 | 3.0E-10 |
| Pathways in cancer | 326 | 116 | 49.01 | 2.37 | 3.1E-20 | 7.0E-18 |
| Phagosome | 153 | 45 | 23.00 | 1.96 | 4.3E-06 | 1.0E-03 |
| Phosphatidylinositol signaling system | 78 | 32 | 11.73 | 2.73 | 2.6E-08 | 5.8E-06 |
| PPAR signaling pathway | 70 | 27 | 10.52 | 2.57 | 1.3E-06 | 3.0E-04 |
| Progesterone-mediated oocyte maturation | 86 | 29 | 12.93 | 2.24 | 1.2E-05 | 2.8E-03 |
| Prostate cancer | 89 | 32 | 13.38 | 2.39 | 9.2E-07 | 2.0E-04 |
| Protein digestion and absorption | 81 | 33 | 12.18 | 2.71 | 1.9E-08 | 4.3E-06 |
| Purine metabolism | 162 | 48 | 24.35 | 1.97 | 1.7E-06 | 4.0E-04 |
| Pyrimidine metabolism | 99 | 29 | 14.88 | 1.95 | 2.0E-04 | 4.5E-02 |
| Regulation of actin cytoskeleton | 213 | 81 | 32.02 | 2.53 | 1.8E-16 | 3.9E-14 |
| Renal cell carcinoma | 70 | 25 | 10.52 | 2.38 | 1.6E-05 | 3.6E-03 |
| Salivary secretion | 89 | 28 | 13.38 | 2.09 | 7.2E-05 | 1.6E-02 |
| Shigellosis | 61 | 21 | 9.17 | 2.29 | 1.0E-04 | 2.3E-02 |
| Small cell lung cancer | 85 | 30 | 12.78 | 2.35 | 3.1E-06 | 7.0E-04 |
| Sphingolipid metabolism | 40 | 17 | 6.01 | 2.83 | 2.7E-05 | 6.1E-03 |
| Starch and sucrose metabolism | 54 | 21 | 8.12 | 2.59 | 1.7E-05 | 3.8E-03 |
| T cell receptor signaling pathway | 108 | 39 | 16.23 | 2.40 | 5.5E-08 | 1.2E-05 |
| Tight junction | 132 | 50 | 19.84 | 2.52 | 1.1E-10 | 2.5E-08 |
| Toxoplasmosis | 132 | 45 | 19.84 | 2.27 | 4.1E-08 | 9.1E-06 |
| Type I diabetes mellitus | 43 | 18 | 6.46 | 2.78 | 2.0E-05 | 4.6E-03 |
| Type II diabetes mellitus | 48 | 23 | 7.22 | 3.19 | 7.4E-08 | 1.7E-05 |
| Vascular smooth muscle contraction | 116 | 56 | 17.44 | 3.21 | 2.9E-17 | 6.5E-15 |
| VEGF signaling pathway | 76 | 30 | 11.42 | 2.63 | 1.9E-07 | 4.3E-05 |
| Vibrio cholerae infection | 54 | 19 | 8.12 | 2.34 | 2.0E-04 | 4.5E-02 |
| Viral myocarditis | 70 | 25 | 10.52 | 2.38 | 1.6E-05 | 3.6E-03 |
| Wnt signaling pathway | 150 | 51 | 22.55 | 2.26 | 5.8E-09 | 1.3E-06 |

C, the number of reference genes in the category; O, the number of genes in the gene set and also in the category; E, expected number in the category; R, the ratio of enrichment, rawP, the p value from hypergeometric test; adjP, the p value adjusted by the multiple test adjustment.
